# Approach-Bias Predicts Development of Cannabis Problem Severity in Heavy Cannabis Users: Results from a Prospective FMRI Study

**DOI:** 10.1371/journal.pone.0042394

**Published:** 2012-09-05

**Authors:** Janna Cousijn, Anna E. Goudriaan, K. Richard Ridderinkhof, Wim van den Brink, Dick J. Veltman, Reinout W. Wiers

**Affiliations:** 1 Department of Psychology, University of Amsterdam, Amsterdam, The Netherlands; 2 Amsterdam Institute for Addiction Research, Department of Psychiatry, Academic Medical Centre, University of Amsterdam, Amsterdam, The Netherlands; 3 Cognitive Science Center Amsterdam, University of Amsterdam, Amsterdam, The Netherlands; 4 Department of Psychiatry, Vriie Universteit Medical Center, Amsterdam, The Netherlands; University of Maryland, College Park, United States of America

## Abstract

A potentially powerful predictor for the course of drug (ab)use is the approach-bias, that is, the pre-reflective tendency to approach rather than avoid drug-related stimuli. Here we investigated the neural underpinnings of cannabis approach and avoidance tendencies. By elucidating the predictive power of neural approach-bias activations for future cannabis use and problem severity, we aimed at identifying new intervention targets. Using functional Magnetic Resonance Imaging (fMRI), neural approach-bias activations were measured with a Stimulus Response Compatibility task (SRC) and compared between 33 heavy cannabis users and 36 matched controls. In addition, associations were examined between approach-bias activations and cannabis use and problem severity at baseline and at six-month follow-up. Approach-bias activations did not differ between heavy cannabis users and controls. However, within the group of heavy cannabis users, a positive relation was observed between total lifetime cannabis use and approach-bias activations in various fronto-limbic areas. Moreover, approach-bias activations in the dorsolateral prefrontal cortex (DLPFC) and anterior cingulate cortex (ACC) independently predicted cannabis problem severity after six months over and beyond session-induced subjective measures of craving. Higher DLPFC/ACC activity during cannabis approach trials, but lower activity during cannabis avoidance trials were associated with decreases in cannabis problem severity. These findings suggest that cannabis users with deficient control over cannabis action tendencies are more likely to develop cannabis related problems. Moreover, the balance between cannabis approach and avoidance responses in the DLPFC and ACC may help identify individuals at-risk for cannabis use disorders and may be new targets for prevention and treatment.

## Introduction

A key question to a better understanding of addiction is why some individuals develop a substance use disorder (SUD) while others do not. Substance use and abuse tend to wax during adolescence and then wane during the transition into adulthood [1], [2]. However, in some individuals substance use escalates and becomes a chronic intermittent substance use disorder. In order to prevent the development of SUDs we need to know more about predictors of the progression of recreational to problematic drug use and from there to drug dependence.

Theoretical models suggest that automatic tendencies to approach rather than to avoid substances of abuse or related stimuli (the so-called approach-bias) may play an important role in the development and persistence of addictive behaviors [3], [4]. It is a natural adaptive tendency to approach what is good and to avoid what is bad, but substance-dependent individuals pathologically approach substances of abuse and the circumstances associated with it, despite awareness of the harmful consequences. During the transition from recreational to compulsive substance use, an imbalance is thought to arise between an approach-oriented motivational system and a regulatory executive system [3], [4], [5]. Through repeated substance use, the motivational system becomes conditioned towards the substance of abuse which can lead to potent and relatively automatic tendencies to substance approach without proper inhibition and thus leading to compulsive drug use [4].

Indeed, a behavioral approach-bias (e.g. faster approach vs. avoid responses) towards substance-related materials has been observed in drug-abusing and drug-dependent individuals compared to non-dependent controls [6], [7], [8], [9], [10], [11], [12]. Moreover, an association has repeatedly been reported between the approach-bias and substance use [9], [13], [14], substance use-related problems [9], and craving [14], [15]. The approach-bias also has been found to predict escalation of cannabis use in heavy cannabis users after a six-month follow-up [7]. Finally, it has been shown that heavy drinkers [12] can be retrained to avoid alcohol and successful retraining predicted improved treatment outcome in alcohol-dependent patients [16]. These findings emphasize the potential of approach-bias as a tool in the prediction of SUDs and as a target for prevention and treatment. However, little is known about neural mechanisms underlying biased approach responses.

Functional Magnetic Resonance Imaging (fMRI) studies on experimental approach and avoidance learning suggest that both approach and avoidance learning recruit the same fronto-limbic network including the striatum, amygdala, insula, anterior cingulate cortex (ACC), and various prefrontal areas [17], [18], showing important overlap with the neurocircuitries involved in addiction [5]. Moreover, brain activity in these fronto-limbic areas appears to increase trial-by-trial during approach and avoidance learning [18]. Together, these areas play a role in evaluating reward value and emotional or motivational salience, integrating affective, cognitive, and motivational processes, establishing action-outcome contingencies, and eventually initiating approach and avoid actions [19], [20], [21].

In these studies on approach and avoidance learning, action outcome contingencies congruent with natural adaptive tendencies are learned; approach under positive reinforcement and avoidance under negative reinforcement. In attempts to parse action from valence, two recent fMRI studies (during which participants pushed and pulled a joystick in response to happy and sad faces) showed that ventral parts of the prefrontal cortex are more active when affect-incongruent (avoid happy, approach sad) compared to affect-congruent actions are performed [22], [23]. Also, irrespective of stimulus valence, the dorsolateral prefrontal cortex (DLPFC) seems to be involved in the distinction between approach and avoid actions [24].

To the best of our knowledge, there are no published studies investigating the neural mechanisms of unbalanced approach and avoidance behavior related to substance use. However, within various fronto-limbic areas, increased avoidance related activity has been associated with higher sub-clinical symptoms of anxiety and thus with excessive avoidance behavior [18], [25]. Therefore, balanced approach and avoid tendencies seem to recruit the fronto-limbic circuitry in a similar way and this suggests that unbalanced, pathological approach tendencies in individuals with a SUD may be reflected by increased approach compared to avoid responses within the fronto-limbic circuitry.

Given the suggested importance of approach-bias in the development of addictive behaviors and its potency as a new target for interventions, the goal of the present fMRI study was to investigate the neural mechanisms underlying cannabis approach and avoidance tendencies. By elucidating the predictive power of neural approach-bias activations for future cannabis use and problem severity, we aimed at identifying new intervention targets. Cannabis is one of the most used illegal substances worldwide, and some 7–8% of heavy cannabis users (defined as using at least ten times per month) meet DSM-IV criteria for cannabis dependence [26], [27], [28]. A growing awareness of the addictive properties of cannabis is accompanied by a growing need for research investigating cannabis abuse and dependence and possible prevention and treatment options.

**Figure 1:**
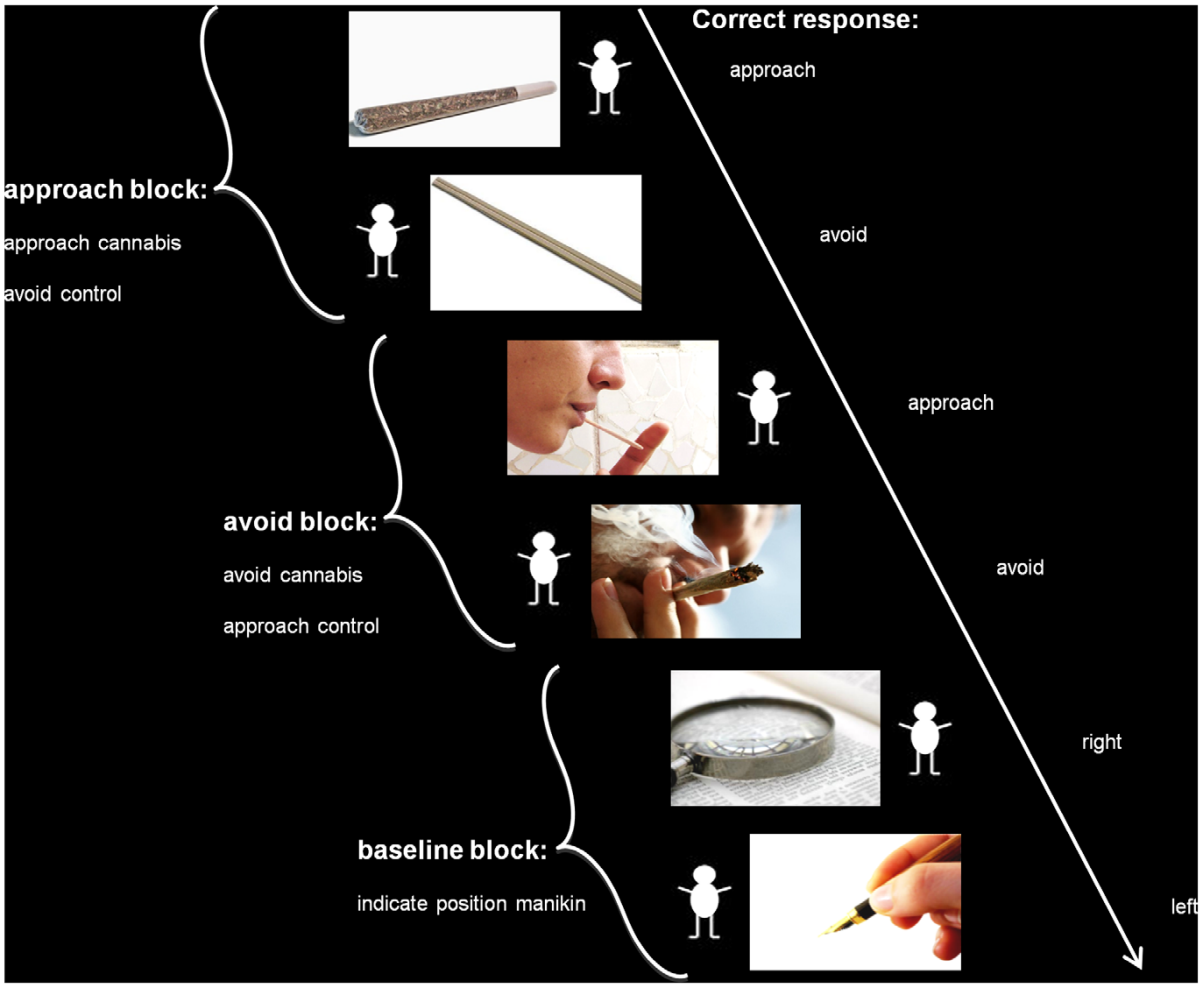
Schematic representation of the Stimulus Response Compatibility Task. The SRC consisted of two approach, avoid, and baseline blocks. Trials consisted of a cannabis or control image with a manikin left or right next to it. Approach-block instructions were to move the manikin towards cannabis images but away from other images. Avoid-block instructions were the reversed. The manikin could be moved left and right by pressing the corresponding left and right response box button. Baseline-block Instructions were to press left or right according to the manikin’s position.

To achieve our goal, neural approach-bias activation patterns were measured with a Stimulus Response Compatibility task (SRC, Figure 1) and compared between 33 heavy cannabis users and 36 matched controls. The SRC has been used successfully to measure behavioral approach-bias in cigarette smokers [6], [10], alcohol drinkers [9], [15], and cannabis users [8]. We expected increased approach-bias activation patterns in fronto-limbic areas among heavy cannabis users compared to controls. Within the group of heavy cannabis users, we further examined how these neural approach-bias activation patterns were related to cannabis use and problem severity at baseline. Finally we examined the predictive effect of neural approach-bias activations for cannabis use and problem severity after a six-month follow-up. Since fMRI costs may outgrow clinical benefits, it was important to verify that the predictive power of neural indices went beyond that of behavioral indices predicting cannabis use and problem severity.

## Materials and Methods

### Ethics Statement

The present study was part of a prospective fMRI study investigating the role of different neurocognitive and neuroimaging factors in the course of drug use in heavy cannabis users [7], [29], [30]. In the current report only participants performing the SRC are described. The medical ethical committee of the Academic Medical Centre of the University of Amsterdam approved the study and all participants signed informed consent prior to participation.

### Participants

Thirty-three heavy cannabis users and thirty-six controls aged 18–25 were recruited through advertisements on the Internet and in cannabis outlets (coffee shops). Groups were matched for age, gender, education, estimated intelligence [31], and alcohol use [32] (Table 1). Heavy cannabis use was defined as using cannabis more than 10 days per month at least for two years and not seeking treatment or having a history of treatment for cannabis use. Participants in the control group smoked less than 50 cannabis joints lifetime and did not use during the last year (5 controls used more than 10 joints lifetime). Drug and alcohol use was controlled for by excluding participants with an Alcohol Use Disorder Identification Test ([AUDIT [32]) score higher than 10, smoking more than 20 cigarettes per day, a positive urine screen for alcohol, amphetamines, benzodiazepines, opioids or cocaine, or using non-cannabinoïd drugs on more than 100 occasions [5 participants >10 occasions, no participant >25 occasions, average time since last occasion was 11.2 months (range 1–36 months)]. Other exclusion criteria were general MRI-contraindications, major physical disorders, and psychiatric disorders, which were assessed with the Mini-International Neuropsychiatric Interview [MINI, Dutch version 5.0.0, 33]. All participants were asked to refrain from alcohol and drug use (except for nicotine and caffeine) in the 24-hours before testing (average self-reported abstinence of cannabis use in heavy users was 1.8 days, SD = 2.3). Although urine analysis of THC metabolites is insensitive to 24-hour abstinence, it increases accuracy of self-reported substance-use [34]. The urine samples were taken to control for recent illicit substance use (all heavy cannabis users scored positive for cannabis use, whereas all controls scored negative). Testing took place in late afternoon. Participants were financially compensated for their participation.

**Table 1:** Sample characteristics.

	Heavy cannabis users		Controls	
	Baseline	Six-month follow-up	Baseline	Six-month follow-up
N (% female)	33 (36)	31 (33)	36 (36)	36 (36)
Age, mean (SD)	21.3 (2.4)	21.7 (2.4)	22.2 (2.5)	22.7 (2.5)
Verbal IQ (Dutch Reading Test), mean (SD)	104.2 (5.4)	–	105.7 (7.1)	–
Alcohol related problems (AUDIT), mean (SD)	6.2 (3.3)	5.6 (3.2)	5.1 (3.4)	5.0 (3.3)
Cigarette smoking (%)	70	63	17^a^	19^a^
Nicotine dependence (FTND), mean (SD)	2.8 (2.4)	2.9 (2.5)	0.5 (1.1)^a^	0.5 (1.0)^a^
Duration cigarette smoking (year), mean (SD)	3.7 (3.6)	3.7 (3.7)	0.6 (1.5)^a^	0.7 (1.7)^a^
Cigarettes per day, mean (SD)	7.0 (7.2)	7.6 (6.9)	1.2 (3.0)^a^	1.2 (2.8)^a^
Cannabis use lifetime (# joints), mean (SD)	1579.5 (1425.0)	1622.5 (1349.1)	5.0 (9.7)	5.6 (10.6)
Cannabis related problems (CUDIT), mean (SD)	12.4 (5.7)	9.5 (6.6)^b^	0 (0)	0.2 (0.5)
Duration heavy cannabis use (year), mean (SD)	2.5 (1.9)	2.9 (1.9)	–	–
Current cannabis use days/week, mean (SD)	4.9 (1.5)	4.9 (2.1)	–	–
Current cannabis use gram/week, mean (SD)	3.0 (2.2)	3.2 (3.0)	–	–
	**Cannabis**	**Neutral**	**Cannabis**	**Neutral**
SRC RT, mean (SD)	793.9 (93.7)	852.1 (128.2)^c^	822.2 (179.7)	893.0 (181.9)^c^
	**Pre-test**	**Post-test**	**Pre-test**	**Post-test**
Cannabis craving (MCQ), mean (SD)	30.3 (12.8)	36.8 (14.0)^d^	12.7 (1.7)	13.1 (2.3)

a *p*<.001 for group comparison.

b *p*<.05 baseline follow-up comparison.

c *p*<.001 approach avoid comparison.

d *p*<.01 pre-test post-test comparison. SD: standard deviation. AUDIT: Alcohol Use Disorder Identification Test [32]. FTND: Fagerström Test for Nicotine Dependence [36]. CUDIT: Cannabis Use Disorder Identification Test [35]. SRC: Stimulus Response Compatibility. RT: reaction time. MCQ: Marijuana Craving Questionnaire [37].

### Questionnaires at Baseline and Follow-up

Problem severity of cannabis use was assessed with the Cannabis Use Disorder Identification Test (CUDIT [35]]. The CUDIT is a screening-instrument for at-risk cannabis use and consists of 10 items on cannabis use-frequency and severity of use-related problems. Severity of nicotine dependence was measured with the Fagerström Test for Nicotine Dependence (FTND [36]). In addition, a detailed history on past and present cannabis and nicotine use was recorded. The short version of the Marijuana Craving Questionnaire [MCQ [37]) was used to assess craving before (pre-test) and after the fMRI-session (post-test). After six months participants were contacted for a telephone interview on present drug use and related problems.

### Event-related SRC Task

Participants performed an fMRI-optimized SRC task during which fMRI-BOLD responses were recorded. The SRC consisted of two approach and two avoid blocks during which full-color cannabis-related images (*n* = 12) and control images (*n* = 12) were presented with a matchstick human-like figure (manikin) left or right next to it (Figure 1). Cannabis images were photos of cannabis, individuals smoking cannabis, and objects for using cannabis. Control images were photos of individuals and objects visually matched to the cannabis images on color and composition. Each image was presented twice per block in semi-random order (max three similar image categories and responses in a row), once with the manikin left and once right, resulting in 48 trials per block. In approach blocks, participants were instructed to move the manikin towards cannabis-related images and away from other (control) images. Instructions were reversed in avoid blocks (avoid cannabis-related images, approach control images). The manikin could be moved left and right by pressing the corresponding button on the left and right response box. After a correct response, the manikin walked towards or away from the image, an incorrect response was followed by a red cross. This feedback lasted 800 ms and the inter-trial interval was jittered between 500 ms and 2000 ms. The task resembles the SCR used by Field et al. (2006), but differed on a number of aspects. First, the manikin was placed next to instead of above and below the image. Second, approach and avoid blocks were presented twice instead of once. Third, after completion of both an approach and avoid block, a baseline block was included during which participants viewed new control images with the instruction to press left or right according to the manikin’s position. These baseline blocks were included to provide an explicit motor baseline. Block-order was ABCBAC or BACABC with the baseline block (C) at the middle and end, counterbalanced over participants. Images were projected on a screen viewed through a mirror attached to the MRI head coil. Average total task time was 12 minutes. Prior to scanning the participants shortly practiced the task outside the scanner.

### Imaging Parameters and Data Pre-processing

A 3T MRI scanner (Philips Intera, Best, The Netherlands) with a phased array SENSE RF eight-channel receiver head coil was used for image acquisition. At start of each scan-session a T1 structural image was acquired (T1 turbo field echo, TR 9.6 s, TE 4.6 ms, 182 slices, slice thickness 1.2 mm, FOV 256×256 mm, in-plane resolution 256×256, flip angle 8°). During the SRC task, BOLD signal was measured with a T2* gradient-echo EPI sequence (TR 2.29 s, TE 30 ms, 38 slices, slice thickness 3 mm, interslice gap 0.3 mm, FOV 220×220 mm, in-plane resolution 96×96, flip angle 80°). Data pre-processing was conducted with FEAT (FMRI Expert Analysis Tool) version 4.1, part of FSL (FMRIB’s Software Library, www.fmrib.ox.ac.uk/fsl). First, non-brain tissue and skull was removed with BET (Brain Extraction Tool). Images were then slice-time aligned, motion corrected, high-pass filtered in the temporal domain (sigma = 50 s), spatially smoothed with a 5 mm full-with-half-maximum Gaussian kernel, and prewhitened [38]. Next, functional data were registered to the participants’ structural image and transformed to MNI (Montreal Neurological Institute) space using FLIRT (FMRIB’s Linear Image Registration Tool).

### Statistical Analysis

Demographics, scores on questionnaires, and SRC reaction times (RTs) were compared between groups with standard univariate analysis of variance procedures. Before analysis of SRC RTs, error trials were removed and RTs below 200 ms, above 2000 ms, and more than 3 SD above mean were removed to correct for outliers. Pearson correlations and hierarchical multiple regression analysis were used to investigate associations between neural cannabis approach responses, craving, measures of cannabis use and problem severity at baseline and six-month follow-up, and cigarette smoking.

fMRI time-series analysis was carried out with FILM (FMRIB’s Improved Linear Model) with local autocorrelation correction. Explanatory variables were created for approach-cannabis, avoid-cannabis, approach-control, avoid-control, and baseline trials by convolving timing parameters with a double gamma hemodynamic response function. Higher-level group analyses of contrast-images were conducted using FLAME (FMRIB’s local analysis of mixed effects) stages 1 and 2. Primary contrast of interest was the cannabis approach-bias, that is approach block (approach-cannabis & avoid-control) > avoid block (avoid-cannabis & approach-control). This contrast enable’s the analysis of differences between cannabis approach and avoidance corrected for differences between control approach and avoidance. Additionally, the four separate condition > baseline contrast were investigated for descriptive purposes. Activity was considered significant if *Z* >2.3, with a whole-brain corrected cluster probability of *p*<.05 [39]. Clusters of activation were localized with the Talairach Daemon database implemented in FSL and the LONI probability atlas [40].

Approach-bias activation patterns were first compared between heavy cannabis users and controls. Second, within heavy cannabis users, multiple regression analyses were performed to investigate associations between approach-bias activation and history of cannabis use, using baseline weekly use (grams), lifetime use (number of joints), duration of heavy use (years), and baseline problem severity (CUDIT) as dependent measures. Third, to assess possible confounding effects of nicotine use, associations between approach-bias activations and FTND scores, duration of cigarette smoking (years), and cigarettes smoked per day were investigated and smoking heavy users were compared to non-smoking heavy users. Fourth, a series of analyses was performed to investigate associations between approach-bias activations at baseline and changes in cannabis use and problem severity after six months. To identify approach-bias activations that predicted changes in cannabis use and problem severity after six months, multiple regression analyses were performed using change in weekly use (follow-up gram per week-baseline gram per week) and change in problem severity (follow-up CUDIT – baseline CUDIT) as dependent variables. Subsequently, a confirmatory hierarchical multiple regression analysis was performed to verify if predictive power of neural indices went beyond that of behavioral indices. For this purpose approach-bias activation in significant clusters was quantified for each participant by extracting the percent BOLD signal change for the approach-bias contrast with Featquery (implemented in FSL).

## Results

### SRC Behavioral Performance

SRC performance was 94% correct (range = 76–100%) with no difference between groups. Overall median RTs did not differ between Groups (*t_67_* = 1.00, *p* = .32, Table 1). RTs were further analyzed using a mixed ANOVA with Group and Block Order (ABCBAC or BACABC) as between-subject factors and Block as within subject factor with two levels [approach (approach-cannabis & avoid-control), avoid (avoid-cannabis & approach-control)]. A main effect of Block, *F*_1, 65_ = 35.91, *p*<.001, *η* ^2^ = .36 did not differ between Groups, *F*_1,65_ = .27, *p* = 0.61, indicating that both Groups were faster during approach compared to avoidance blocks. This general approach-bias was, however, modulated by Block Order, *F*_1,65_ = 13.30, *p* = .001, *η* ^2^ = .17: the RT-difference between approach and avoidance was largest for participants starting with an avoid block (*t_67_* = 3.72, *p*<.001, *d* = .90). To account for block-order effects, all fMRI analyses were controlled for Block Order by entering it as an additional covariate into the regression model for the BOLD signal.

### SRC fMRI

**Figure 2:**
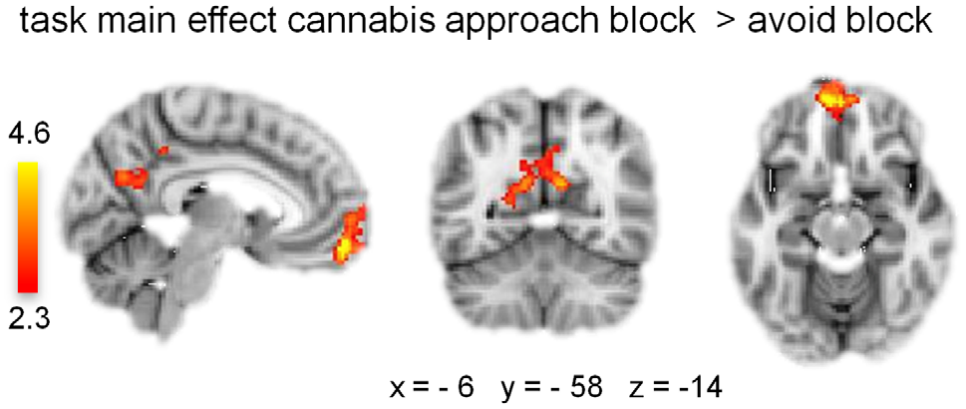
Main effect of approach vs. avoid blocks over groups. Clusters of significant activation in ventral medial frontal gyrus and posterior cingulate gyrus (*Z* >2.3, whole-brain cluster-corrected at *p*<0.05) are overlaid on a standard MNI brain. Right side of the brain is depicted at right side.

Approach-bias activations [approach block (approach-cannabis & avoid-control) > avoid block (avoid-cannabis & approach-control)] were observed across groups in the ventromedial prefrontal cortex and posterior cingulate gyrus (Figure 2, Table 2). In contrast to our hypothesis, no significant differences were observed in approach-bias activations between groups.

**Table 2:** Cannabis approach-bias activations: main task effect across groups and correlation lifetime cannabis use and change problem severity (CUDIT-scores) at six-month follow-up in heavy cannabis users.

Clustersize	Brain region	Hemisphere	MNI coordinates			*Z*_max_
(voxels)			*x*	*y*	*z*	
***Main effect approach vs. avoid***						
787	Ventral medial frontal gyrus	R/L	−6	56	−14	4.55
578	Posterior cingulate gyrus	R/L	10	−56	20	3.50
***Lifetime cannabis use positive correlation***						
3801	Parahippocampal gyrus	L	−14	−34	−8	3.96
	Amygdala	R	16	−4	−18	3.40
	Occipital cortex	L/R	−6	−58	2	3.62
	Cerebellum	R	34	−40	−30	3.76
1598	Cerebellum	L	−34	−72	−28	3.83
2221	Insula	R	38	10	2	3.78
	Inferior frontal gyrus, pars opercularis	R	52	16	4	3.16
1082	Medial frontal gyrus	L/R	6	56	−4	3.21
686	Precuneus	R	4	−62	60	3.83
495	Supramarinal gyrus, BA 40	L	−54	−48	24	3.41
***Change CUDIT negative correlation***						
413	Dorsolateral prefrontal cortex	R	36	32	36	3.54
746	Anterior cingulate cortex	L/R	−8	42	18	3.34

L, left; R, right; MNI, Montreal Neurological Institute; BA, Brodmann Area; MNI coordinates and *Z*-scores of local maxima are shown for each cluster; Statistical threshold: *Z* >2.3, whole-brain cluster-corrected at *p*<0.05.

**Figure 3:**
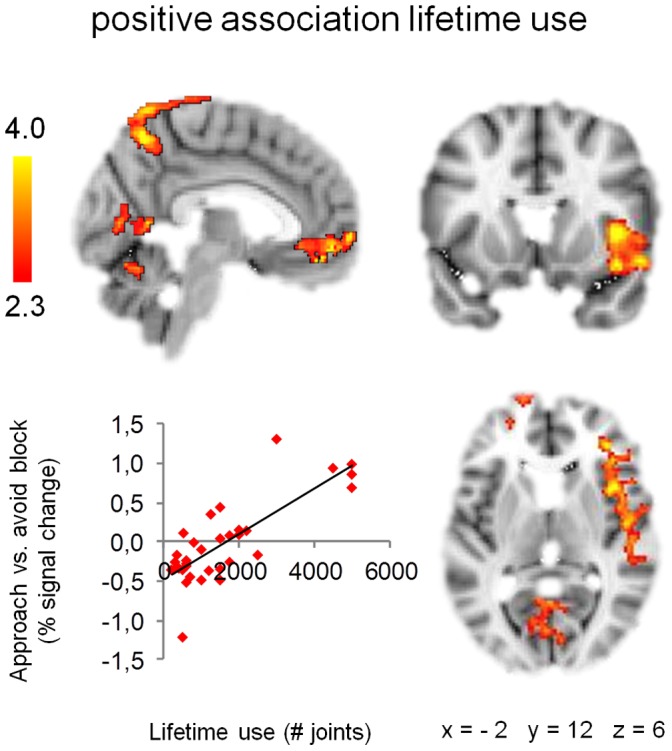
Association approach-bias activation patterns and lifetime cannabis use. Approach-bias activation patterns in right insula, medial frontal gyrus, precuneus, cerebellum, and occipital cortex are positively associated with lifetime cannabis use among heavy cannabis users. Scatter plot shows association lifetime use and average percent signal change extracted from significant clusters. Clusters of significant activation (*Z* >2.3, whole-brain cluster-corrected at *p*<0.05) are overlaid on a standard MNI brain. Right side of the brain is depicted at right side.

Within the group of heavy cannabis users, a significant positive relation was observed between lifetime cannabis use and approach-bias activations in various fronto-limbic areas including the right amygdala, right insula, right inferior frontal gyrus, bilateral ventromedial prefrontal gyrus, and left parahippocampal gyrus. Lifetime cannabis use further predicted activation in the left supramarginal gyrus, right precuneus, bilateral cerebellum, and bilateral occipital cortex (Figure 3, Table 2). No significant relations were found between approach-bias activations and baseline problem severity, baseline weekly cannabis use, or duration of heavy cannabis use.

Heavy cannabis users scored higher on measures of nicotine use [smokers (%), *X* ^2^ = 19.87, *p*<.001]; nicotine dependence [FTND [36], *t_67_* = 5.43, *p*<.001; smoking duration (years), *t_67_* = 4.83, *p*<.001, and cigarettes per day, *t_67_* = 4.39, *p*<.001; Table 1]. Moreover, all measures of nicotine use correlated positively with duration of heavy cannabis use (*r* >.40, *p*<.023). However, neural approach-bias activation patterns did not differ between smoking and non-smoking heavy cannabis users and did not co-vary with nicotine dependence, smoking duration, and number of cigarettes smoked per day.

### Neural and Behavioral Predictors of Problem Severity after Six Months

Six months after baseline a 97% follow-up rate was achieved (two non-responders among the heavy cannabis users). In accordance with normative trajectories of cannabis use in young adults [2], average cannabis problem severity decreased in heavy cannabis users (*t_30_* = 2.4, *p* = .022, Table 1). Cannabis use frequencies and measures of alcohol and nicotine use did not change in heavy cannabis users or controls (Table 1).

**Figure 4:**
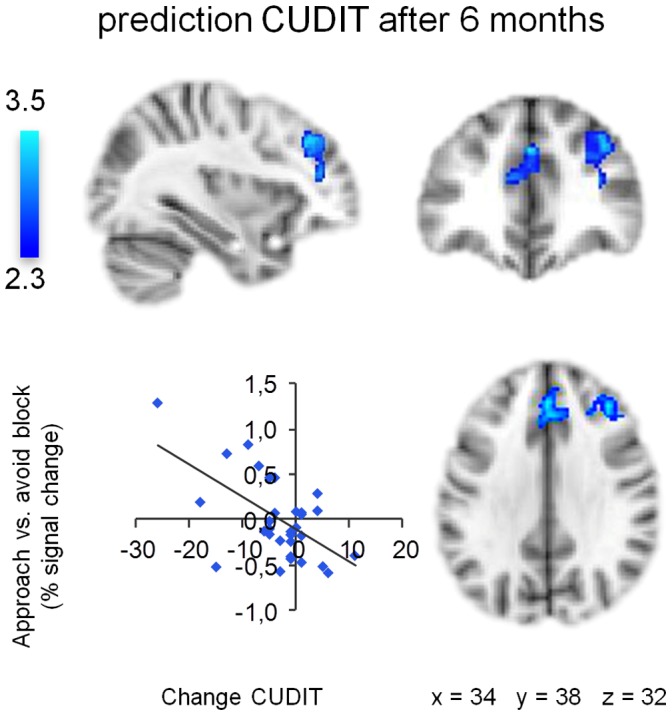
Association approach-bias activation patterns and change in cannabis problem severity. Approach-bias activation patterns in right dorsolateral prefrontal cortex and anterior cingulate cortex are negatively associated with changes in cannabis problem severity (CUDIT-scores) among heavy cannabis users at six-month follow-up. Scatter plot shows association change in CUDIT-scores and average percent signal change extracted from significant clusters. Clusters of significant activation (*Z* >2.3, whole-brain cluster-corrected at *p*<0.05) are overlaid on a standard MNI brain. Right side of the brain is depicted at right side.

Within the group of heavy cannabis users, a negative association was observed between approach-bias activations in the DLPFC and ACC and changes in problem severity: the weaker the approach-bias activation in the right DLPFC and ACC the larger the increase in problem severity (Figure 4, Table 2). No relations were found between approach-bias activations and changes in weekly cannabis use.

When correlating change in problem severity with the behavioral approach-bias derived from the RT data and self-reports of substance use and craving at baseline, a significant relation was also observed between change in problem severity and session-induced craving (*r* = .51, *p* = .004), but not with the behavioral approach-bias (*p* = .80), baseline weekly cannabis use (*p* = .85), lifetime cannabis use (*p* = .25), duration of heavy cannabis use (*p* = .34), nicotine dependence (*p* = .88), smoking duration (*p* = .82), cigarettes per day (*p* = .48), alcohol use and problems (*p* = .31), pre-test craving (*p* = .31), and post-test craving (*p* = .06).

To verify if approach-bias activation in the DLPFC and ACC explained unique variance in future problem severity beyond variance explained by session-induced craving at baseline, a confirmatory hierarchical multiple regression analysis was performed. In the hierarchical regression model, baseline CUDIT-scores and session-induced craving were entered first, before the average DLPFC/ACC approach-bias index. Preliminary analyses indicated no violation of the assumption of normality, linearity, multicollinearity, and homoscedasticity (maximum Cook’s distance  = 0.61, maximum standardized residual  = 2.3). The total variance explained by the final model amounted to 36% (*F*_3,27_ = 5.01, *p = *.007, Table 3). Baseline CUDIT-scores (*p* = .018) and session induced craving (*p* = .026) were both significant predictors in the first step and together explained 24% of the variance in CUDIT-scores six months later. After correction for variance explained by session-induced craving, the DLPFC/ACC approach-bias index explained an additional 12% of the variance in CUDIT-scores six months later (*F* change_1,27_ = 5.02, *p* = .032). Participants with a higher DLPFC/ACC approach-bias index had lower CUDIT-scores after six months. In the final model, the baseline CUDIT-score remained a significant predictor (*p* = .002), whereas session-induced craving dropped out (*p* = .064).

To investigate the extent to which the predictive relationship between the DLPFC/ACC approach-bias index and cannabis problem severity was driven by approach or avoid responses we performed a secondary regression analysis. In this regression model, the DLPFC/ACC approach-bias index was replaced with average DLPFC/ACC activity for approach and avoid cannabis trials vs. active baseline. Approach cannabis (*p* = .003) and avoid cannabis (*p* = .002) trials both uniquely explained variance in CUDIT-scores six months later and improved the previous model by explaining 26% of the variance (*F* change_2, 26_ = 6.67, *p* = .005). The total variance explained by the model amounted to 50% (*F*_4,26_ = 6.41, *p = *.001; Table 3). Participants with higher DLPFC/ACC activity during approach cannabis trials had lower CUDIT-scores whereas higher activity during avoid cannabis trials was associated with increased CUDIT-scores after six months.

## Discussion

The goal of this fMRI study was two-fold: first, to investigate the neural basis of cannabis approach and avoid responses in heavy cannabis users, and, second, to assess the predictive power of these neural approach-bias activations for future cannabis use and problem severity. In contrast to our hypothesis, no brain areas showed greater approach-bias activations in heavy cannabis users compared to controls. However, within the heavy cannabis users, approach-bias activations in various fronto-limbic areas were more pronounced with increased lifetime use, which is in line with previous studies on human approach-avoidance learning [18]. Most important, beyond self-reports of session-induced craving, approach-bias activation in DLPFC and ACC predicted problem severity after six months. This novel finding underlines the potential of neural approach-bias activations as predictors of cannabis problem severity and identifies the DLPFC and ACC as loci for targeted interventions.

Stronger DLPFC and ACC activation during cannabis approach vs. avoid was related to decreases in cannabis related problem severity. The DLPFC is involved in regulatory self-control (i.e., providing top-down guidance to more basal cognitive processes supported by networks elsewhere in the brain [41]), and hypoactivation has been linked to poor decision-making in dependent cannabis users [42]. The ACC is involved in evaluative control (i.e., monitoring one’s performance and assessing salience of motivational information [21]) and has been linked to deficient error monitoring associated with substance abuse [43]. The DLPFC and ACC together are thought to play an important role in appropriately adjusting behavior in conflicting situations [44], [45], which may be critical to successfully resist substance use. The observed prospective negative association between DLPFC/ACC activation and future cannabis related problems may then reflect, the fact that those heavy cannabis users with a well-developed capacity to evaluate and regulate their drug use are more likely to reduce or control their cannabis use, rather than the presence of problem severity per se.

**Table 3:** Hierarchical multiple regression analysis for variables predicting cannabis problem severity (CUDIT-scores) at six-month follow-up in heavy cannabis users (n = 31).

	B	SE B	*β*
**Step 1**: Change *R* ^2^: 0.24*			
CUDIT baseline	0.49	0.20	0.44*
Session-induced craving	0.26	0.11	0.41*
**Step 2a**: Change R^2^: 0.12*			
CUDIT baseline	0.69	0.21	0.62*
Session-induced craving	0.20	0.10	0.33
DLPFC/ACC approach bias	−6.00	2.68	−0.41*
**Step 2b**: Change R^2^: 0.26**			
CUDIT baseline	0.65	0.18	0.58***
Session-induced craving	0.18	0.10	0.29
DLPFC/ACC approach cannabis	−10.10	3.10	−0.71**
DLPFC/ACC avoid cannabis	8.50	2.42	0.75**

* *p*<.05, **p<.01, and ***p<.001. Model step 1 and 2a *R* ^2^: 0.36**, adjusted *R* ^2^ 0.29. Model step 1 and 2a *R* ^2^: 0.50***, adjusted *R* ^2^ 0.42. SE: standard error. CUDIT: Cannabis Use Disorder Identification Test. DLPFC: dorsolateral prefrontal cortex. ACC: anterior cingulate cortex.

Interestingly, it has been shown that alcohol-dependent patients can be retrained to avoid alcohol and that successful retraining improved treatment outcome [16]. Recent work in our lab showed that this improvement is probably mediated by increased control over alcohol approach and avoidance responses rather than decreased strength of automatic appetitive approach tendencies. Also, the present findings indicate that both approach and avoidance tendencies towards cannabis explain unique variance in the change in problem severity. These findings underline the notion that the approach-bias observed in individuals with a SUD does not merely reflect sensitized and conditioned bottom-up drug-approach tendencies. Instead, control (or the lack thereof) over approach and avoidance behavior in a substance-specific context could be the primary mediator of the relation between approach-bias, continued substance use and substance use-related problems. Future research efforts should be aimed at confirming these inferences.

Approach as well as avoidance responses engage activation in fronto-limbic areas, with considerable overlap between these areas [17], [18]. We hypothesized that a cannabis approach-bias would result in increased activation for cannabis approach compared to avoidance in these fronto-limbic areas. Across groups, we found such differences in ventromedial prefrontal and posterior cingulate cortex. In contrast to our hypothesis, these activations were very similar for controls and heavy cannabis users, suggesting that a history of cannabis use in the present sample did not suffice to alter activation of these areas. However, within the group of heavy cannabis users, lifetime cannabis use predicted approach-bias activations in various fronto-limbic areas including the amygdala, insula, inferior frontal gyrus, medial frontal gyrus, and parahippocampal gyrus but also visual areas, precuneus, and the cerebellum. Short-term experience-related increases have been observed in all these areas during approach and avoidance learning [18]. The observed correlation with lifetime use may indicate that increased front-limbic activity extends beyond short-term rapid learning processes and probably reflect increased salience and motivation for cannabis over time. Moreover, the lack of an association with cannabis problem severity and weekly cannabis use suggests that the increased fronto-limbic response to cannabis approach relative to avoidance may be a function of lifetime cannabis exposure, rather than cannabis problem severity or the direct (sub)acute effects cannabis use. Given the relatively young age and short duration of heavy cannabis use in the present sample, the association with lifetime use raises the hypothesis that group differences could be expected in more long-term cannabis users compared to controls. This further suggests that all cannabis users could develop increased salience and motivation for cannabis over time.

We have previously shown, using the same sample as in the present study, that heavy cannabis users had an approach-bias for cannabis related materials as measured with a different joystick approach-avoidance task. Moreover, the approach-bias predicted absolute levels of cannabis use after six months [7]. The current study contributes the important novel finding that approach-bias activation in DLPFC and ACC explain unique variance in future cannabis use-related problems. However, the lack of differences in approach-bias activation patterns and RTs might suggest a limitation in the construct validity of the task. To the best of our knowledge, this is the first study to use the SRC combined with fMRI. Further studies are needed to verify if the SRC is a reliable task to measure the neural mechanisms underlying approach and avoidance behavior.

Some potential limitations must be taken into account. First, there were more smokers among heavy cannabis users and almost all cannabis users (90%) smoked cannabis combined with tobacco (by far the most common form of cannabis use in the Netherlands [46]). Since the heavy cannabis users were relatively light smokers and nicotine use was not significantly associated with approach-bias activations in heavy cannabis users, it is unlikely that nicotine use accounts for the observed effects. However, duration of heavy cannabis use correlated with nicotine use and we cannot exclude potential confounding effect of nicotine use. A post-hoc hierarchical regression analysis was performed with FTND-score as additional covariate to verify if DLPFC and ACC activity still predicted cannabis problem severity after correction nicotine dependence. This analysis showed that nicotine dependence did not affect the predictive relationship between DLPFC and ACC activity and cannabis problem severity (DLPFC/ACC approach-bias index *p* = .037). Nevertheless, it may still be worthwhile to include a group of cannabis naive cigarette smokers in future studies in order to better distinguish cannabis from nicotine effects. Also, it should be mentioned that we excluded potential participants if they had a history of psychiatric disorder; a less stringently selected but more ecologically valid control group may display considerable comorbid externalizing disorders. Therefore, the extent to which the results generalize to all heavy cannabis users remains to be tested.

In summary, the current fMRI study is the first to investigate the neural mechanisms underlying the approach-bias in SUDs. In addition to and independent from self-reported clinical characteristics (including craving), cannabis-specific approach-bias activation in the DLPFC and ACC predicted the course of cannabis related problemsin heavy cannabis users. These findings highlight the importance of the approach-bias in maintenance of addictive behaviors and support a specific role for DLPFC and ACC functionality as a biomarker in the prediction of problem severity and as new loci for targeted prevention and treatment.
